# Recurrence of digital papillary invasive adenocarcinoma

**DOI:** 10.1093/jscr/rjad296

**Published:** 2023-06-28

**Authors:** Nadeem Chaudhry, Abid Qureshi, Rutva Shah, Asad Razzaq, Brittni J Clopton, Romulo Genato, Philip Xiao

**Affiliations:** Department of Plastic Surgery, The Brooklyn Hospital Center, Icahn School of Medicine, Brooklyn, NY, USA; Department of Surgery, The Brooklyn Hospital Center, Icahn School of Medicine at Mount Sinai, Brooklyn, NY, USA; School of Medicine, St. George’s University, School of Medicine, Great River, NY, USA; School of Medicine, St. George’s University, School of Medicine, Great River, NY, USA; School of Medicine, American University of the Caribbean, Miramar, FL, USA; Department of Surgery, The Brooklyn Hospital Center, Icahn School of Medicine at Mount Sinai, Brooklyn, NY, USA; Department of Pathology, The Brooklyn Hospital Center, Icahn School of Medicine at Mount Sinai, Brooklyn, NY, USA

## Abstract

Adenocarcinoma is extremely uncommon in the digits with an incidence of 0.08 per 1 000 000 people per year, known as digital papillary adenocarcinoma (DPA). This disease is commonly described pathologically as malignancy of the sweat glands. The fundamental histologic characteristics of DPA are the presence of papillary projections in cystic spaces in a multinodular tumor lined by epithelial cells. DPA are often delayed in diagnosis because of either misdiagnosis for benign lesions or underreporting, which can contribute to poor prognosis and metastasis. This report serves to present a case of recurrence observed in primary digital adenocarcinoma and to bring awareness to the topic as concrete management continues to develop.

## INTRODUCTION

Adenocarcinoma is a type of malignant cancer of glandular epithelial tissue most commonly found in the breasts, lungs, prostate and gastrointestinal tract [1]. Despite its frequent occurrence in the other areas of the body, digital papillary adenocarcinoma (DPA) is extremely rare. One of the first few documented reports of malignant adenocarcinoma found in the finger was in 1984 by Heldwig [2]. Malignancy in the digital region tends to have a delayed diagnosis likely because of being mistaken as a benign lesion and absence of suspicion for primary cancer, which can have fatal consequences considering a local recurrence of 50% and metastasis of 14% [3]. Most of the recurrence after initial excision on average is between 2 months and 9 years, highlighting the importance of obtaining diagnosis and appropriate treatment planning [4]. Because of the rarity and inconspicuous nature of primary DPA, it is vital to keep it as a differential when working with digital lesions. This report serves to present a case of recurrence observed in primary digital adenocarcinoma and to bring awareness to the topic as concrete management continues to develop.

## CASE REPORT

A 45-year-old male with hypertension presented with a finger nodule on the left fifth digit. Patient had a history of soft tissue tumor in the same location that was excised 1-year prior and was found to be positive for adenocarcinoma on pathology. On initial visit, the patient was experiencing tenderness at the site where the nodule was located but there were no associated motor or sensory deficits. Physical examination revealed a solitary 2 × 1 cm nodule on the dorsal aspect of the digit, suspicious for soft tissue tumor. Scarring of the skin was noted. Patient was taken for tumor resection, revealing adenocarcinoma with involvement of one of the lateral margins. Patient was subsequently taken back to the operating room for re-excision and full thickness skin grafting using the left groin, for which pathology came back negative. There were no postoperative complications.

## PATHOLOGY

**Figure 1:**
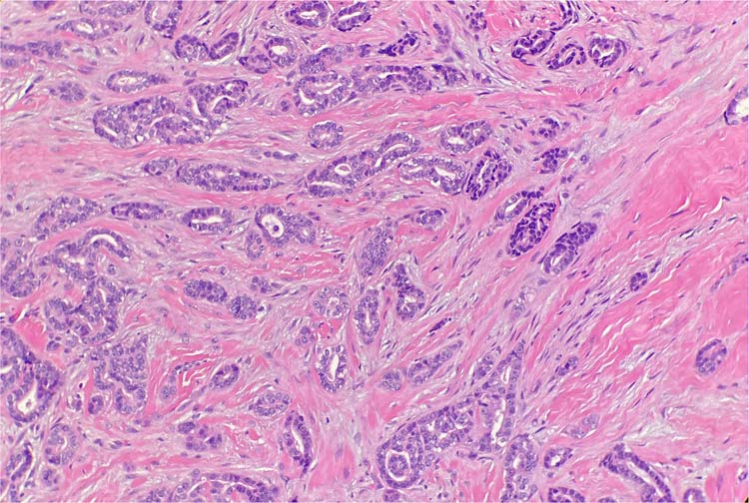
Microscopic examination reveals infiltrative malignant glands with angulated contours. H&E ×20.

Received are multiple fragments of pink tan soft tissue. Microscopic examination reveals infiltrative growth pattern, presence of atypia and lack of myoepithelial cells (Fig. 1). This supports the diagnosis of a well-differentiated sclerosing ductal adenocarcinoma. Patient does not have a history of a prior adenocarcinoma elsewhere; the findings are consistent with a primary cutaneous adenocarcinoma of sweat/duct origin.

## DISCUSSION

Palpable nodules found in the finger can include benign and malignant lesions, including cysts, acrochordon, nevi, basal cell carcinomas, squamous cell carcinomas and melanomas [5]. Adenocarcinoma is a malignant neoplasm of epithelial cells, an adenomatous transformation because of potential genetic factors or external environmental stressors such as harmful prolonged sun exposure, environmental pollutants, ionizing radiation, chemical carcinogens and other work-related exposures [6]. It is extremely uncommon in the digits with an incidence of 0.08 per 1 000 000 people per year [7].

DPA in the past has been referred to as ‘aggressive digital papillary adenocarcinoma’ throughout various literary documents. This disease is commonly described pathologically as malignancy of the sweat glands that occur mostly on the hands and feet in a 3:1 ratio. DPA are often delayed in diagnosis because of either misdiagnosis for benign lesions or underreporting, which can contribute to poor prognosis and metastasis. The fundamental histologic characteristics of DPA are the presence of papillary projections in cystic spaces in a multinodular tumor lined by epithelial cells [8]. This tumor can also be described to have locally aggressive behavior with poor glandular distribution and necrosis with invasion into blood vessels and soft tissue [9].

Our case of DPA highlighted not only the primary malignancy but also the case of recurrence. The histopathologic findings in the patient we presented demonstrated a well-differentiated invasive adenocarcinoma seen to be infiltrating into the reticular dermis without lymphovascular invasion. According to our patient’s age at onset, negative family history, recovery after tumor removal and histopathologic findings, we were able to rule out all variations of the above-mentioned benign and malignant disorders and instead confirm the diagnosis of adenocarcinoma of the finger.

Given the rarity of the condition and no current outlines of management of DPA, the treatment choice is dependent on histologic findings and invasion, usually with surgical removal and wide margins leading to less probability of recurrence and metastatic rates [10]. It has been discussed whether wide local excision or complete digital amputation is appropriate to obtain lower recurrence rates. Because of the tumor’s ability to quickly metastasize and seed elsewhere in the body, being able to perform a wide local excision with negative margins may not stop the disease from progressing. In one study, 14 of the 57 patients proceeded with wide local excision in hopes to save the diseased digit and avoid local spread. However, this study found a recurrence rate of 57.1% in the wide local excision patients [4]. With recurrence rates possibly being as high as 50%, future studies are being conducted in order to establish a management strategy for DPA. Some of these include chemotherapy for unresectable metastases and sentinel node biopsy for staging and documenting, which patients may be at higher risk for systemic metastasis [9].

Though these may be new avenues of research and study, none listed above have proven to be a definitive treatment option for DPA. Therefore, long-term follow-up with a multidisciplinary team is recommended as current management [11].

## CONCLUSION

Because of the prognostic and invasive nature of DPA, it could be argued that close postoperative monitoring is necessary. Additionally, monitoring for potential metastatic spread alongside a multidisciplinary team is of the utmost importance. This disease poses uncharted land for the field of medicine and surgery, lending itself to the opportunity for development of new management strategies for DPA, further exploration on potential pathologic markers contributing to the development of these cancers, as well as the role of possible immunobiologic modulators for treatment. As more cases are being documented, the increased exposure and the more opportunity we have to help those affected by this disease. We look forward to exploring the pathology as a potential differential in patients experiencing masses on the hand.
